# *MUTYH* Gene Polymorphisms as Risk Factors for Rheumatoid Arthritis

**DOI:** 10.1155/2015/893796

**Published:** 2015-07-27

**Authors:** Yung-Jen Kung, Kun Shi Tsai, Chung-Ming Huang, Hui-Ju Lin, Ter-Hsin Chen, Yu-An Hsu, Ching-Yao Chang, Yong-San Huang, Lei Wan

**Affiliations:** ^1^Department of Veterinary Medicine, National Chung Hsing University, Taichung 40227, Taiwan; ^2^Department of Biological Science and Technology, National Chiao Tung University, Hsinchu 30010, Taiwan; ^3^Graduate Institute of Integrated Medicine, China Medical University, Taichung 40402, Taiwan; ^4^Division of Immunology and Rheumatology, Department of Internal Medicine, China Medical University Hospital, Taichung 40402, Taiwan; ^5^Department of Ophthalmology, China Medical University Hospital, Taichung 40402, Taiwan; ^6^Graduate Institute of Veterinary Pathobiology, National Chung Hsing University, Taichung 40227, Taiwan; ^7^Department of Life Science, National Tsing Hua University, Hsinchu 30013, Taiwan; ^8^Department of Medical Research, China Medical University Hospital, Taichung 40402, Taiwan; ^9^Department of Biotechnology, Asia University, Taichung 41354, Taiwan; ^10^School of Chinese Medicine, China Medical University, No. 91, Hsueh-Shih Road, Taichung 40402, Taiwan; ^11^Department of Gynecology, China Medical University Hospital, Taichung 40402, Taiwan

## Abstract

*Objectives.* MUTYH glycosylase involved in DNA repair pathways may be associated with the risk of autoimmune diseases such as rheumatoid arthritis (RA). Therefore, the association between polymorphisms in the *MUTYH* gene and RA was evaluated. *Methods*. We recruited 192 RA patients and 192 healthy subjects in Taiwan. The 4 *MUTYH* polymorphisms (rs3219463, rs3219476, rs3219489, and rs3219493) were detected and haplotype analysis was performed using the Bayesian method. The genotype and allelic frequency distributions of the polymorphisms in both RA patients and healthy patients were compared by the chi-square test. *Results*. Comparison of the genotype/allele frequencies between individuals with RA and the control groups revealed significant differences in 2 *MUTYH* gene polymorphisms, rs3219463 and rs3219476. After we performed a haplotype-specific analysis, the haplotypes Ht6-GTGC and Ht8-GGCG had lower presenting rates in RA patients than in the control groups. Furthermore, the genotype frequency of rs3219463 G/  was significantly increased among patients with immunoglobulin M rheumatoid factors, whereas that of rs3219476 was not. *Conclusion*. We demonstrated that the rs3219463 and rs3219476 polymorphisms in RA patients from a Taiwan Chinese population were associated with disease susceptibility. These data indicate that the *MUTYH* gene may play a role in the progression of RA.

## 1. Introduction

Rheumatoid arthritis (RA) is a chronic autoimmune disease that can lead to deformities and severe disability because of irreversible damage to the tendons, joints, and bones. Although RA is a common type of arthritis with a prevalence of 1%, the pathogenesis is still unknown [1, 2]. RA can be considered to be a complex genetic disease and many “disease-risk” genes or “disease-protective” genes may be involved [3, 4].

Numerous studies have reported an association between* HLA-DR* gene polymorphisms [4] and RA, and CD4 T cells play important roles in the development of RA [5]. In addition, the chronic inflammatory process of RA is mediated through the cytokines network, which induces the expression of some destructive enzymes, such as matrix metalloproteinases in the cellular synovial tissue of joints [1]. Although there is no conclusive evidence, there are hints that genes and enzymes of DNA repair may be involved in some types of autoimmune diseases. It had been suggested that the T cells in RA patients fail to produce sufficient transcripts and proteins of the DNA repair kinase* ataxia telangiectasia mutated* (*ATM*) gene [6]. Therefore, we are interested in whether there are other mechanisms of DNA repair related to the etiology of RA. It has been reported that p53 mutations have been observed in microdissected RA synovial tissue [7]. This strongly suggests that somatic DNA mutations induced by oxidative stress enhance the transformation of cells involved in the invasive excess growth of the RA joints and that a loss of genomic stability is one of the key mechanisms related to the severity of RA. Additionally, because inflammatory responses are the main characteristics of RA [4], we have focused on the deficient DNA repair enzymes related to severe or chronic inflammation.

Inflammation can elicit a variety of cellular signals and induce the formation of reactive oxygen species (ROS), which threaten the integrity of cellular DNA. The oxidized base 7,8-dihydroxy-8-oxoguanine (8-oxo-G) is one of the mutagenic products of oxidative DNA damage; however, misincorporated 8-oxo-G can be excised by the base excision repair (BER) pathway [8]. MUTYH glycosylase, which is encoded by* MUTYH* (*mutY Homolog* (*Escherichia coli*)), is involved in this pathway. When incorrect pairing, 8-oxo-G·A, occurs, MUTYH glycosylase intercepts the 8-oxo-G·A base pair and removes the inappropriate A base and provides an opportunity for creation of an 8-oxo-G·C substrate for repair by 8-oxo-G glycosylase (OGG1) [9–11]. MUTYH glycosylase directly interacts with various proteins involved in other DNA repair pathways [12], and defective MUTYH function is associated with various forms of cancer, including lung cancer [13], gastric cancer [14], head and neck squamous cell carcinomas [15], and colorectal cancer (CRC) [16]. Because there is evidence which suggests that ROS-induced DNA damage contributes to tumor development in a mouse model of chronic inflammation [9], we sought to determine whether mutations in* MUTYH* are risk factors of RA.

We hypothesized that genetic variations in* MUTYH* (in its promoter region, introns, or exons) may be associated with the risk of autoimmune diseases such as RA. Therefore, we analyzed the genetic and allelic frequencies of 4 polymorphisms in* MUTYH* and the haplotypes among RA patients and healthy controls in the Taiwan Chinese population. We also compared the genotypes and haplotypes among RA patients with various clinical variables to investigate whether a relationship exists between* MUTYH* polymorphisms and the clinical manifestation of RA.

## 2. Methods

### 2.1. Patients

The study subjects including a total of 192 patients with RA and 192 healthy subjects were recruited from China Medical University Hospital in Taiwan. Patients with RA according to the revised America College of Rheumatology criteria were enrolled [17]. Nephelometry was used to detect rheumatoid factor (RF). Values ≧ 30 IU/mL were defined as positive. The presence or history of extra-articular manifestations in patients with RA was recorded [18]. The healthy control from the general population was selected from health examination. All individuals' samples were collected by venipuncture for genomic DNA isolation. Informed consent was obtained from all participants and was approved by the local Ethics Committee.

### 2.2. Genomic DNA Extraction and Genotyping

Genomic DNA was prepared from peripheral blood according to standard protocols of the DNA extraction kit (Qiagen, Valencia, CA, USA). The four* MUTYH* single nucleotide polymorphisms (SNPs) (rs3219463, rs3219476, rs3219489, and rs3219493) were detected by a polymerase chain reaction-restriction fragment length polymorphism technique (PCR-RFLP). PCRs for* MUTYH* gene polymorphisms were carried out in a 50 *μ*L reaction mixture containing 50 ng of genomic DNA, 2 to 6 pmole of each primer, 1 × Taq polymerase buffer (1.5 mM MgCl_2_), and 0.25 U of AmpliTaq DNA polymerase (Applied Biosystems). The primers, PCR conditions, and restriction enzyme cutting sites used to determine* MUTYH* polymorphisms were listed in Table 1.

### 2.3. Haplotype Analysis

Haplotypes were inferred from unphased genotype data using the Bayesian statistical method available in the software program Phase 2.1 [19, 20]. All four SNPs were analyzed with the Phase 2.1 software and the population data were divided into 12 groups, from Ht1 to Ht12. A linkage disequilibrium (LD) map was constructed using Haploview software (version 4.2; http://www.broadinstitute.org/scientific-community/science/programs/medical-and-population-genetics/haploview/haploview) to estimate the correlation coefficient between those SNPs.

### 2.4. Statistical Analysis

The chi-square test was utilized to evaluate the Hardy-Weinberg equilibrium in genotypic distributions. Also, the genotype frequency and allelic frequency distributions of the polymorphisms in both RA patients and healthy subjects were compared by the chi-square method (2 × 3 table for genotype frequency; 2 × 2 table for allele frequency) by using the SPSS Version 10.0 software (SPSS Inc., Chicago, IL, USA). Results were considered significant when the *P* values were <0.05. The Bonferroni method was used to correct for multiple testing. The odd ratios (OR) were also calculated from the genomic and allelic frequencies, with a 95% confidence interval (95% CI).

## 3. Results

### 3.1. Demographic Characteristics of Control Subjects and RA Patients

A case and control association study was performed. As shown in the Supplementary Table  1 available online at http://dx.doi.org/10.1155/2015/893796, there are no significant differences in gender and age between control subjects and RA patients. Within the 192 RA patients, 151 people were female and the mean age was 53.4 years. In the healthy controls, 151 people were female and the mean age was 50.5.

### 3.2. Allele and Genotype Frequency of* MUTYH* Polymorphisms

Of the four tested SNPs, three SNPs were found to be in Hardy-Weinberg equilibrium (*P* value > 0.05); however, the genotype distribution of the rs3219476 was not compatible with Hardy-Weinberg equilibrium. The genotype, allele, dominant, and recessive distributions of the 4 polymorphisms are shown in Table 2.

Comparison of the genotypes between individuals with RA and the control groups revealed significant differences in the frequency of 2* MUTYH* gene polymorphisms, rs3219463 and rs3219476. The *P* values were corrected for multiple comparison by Bonferroni correction, represented as *cP* value. For the rs3219463 G/A polymorphism, the *cP* value was 0.00024 and the odds ratio (OR) was 1.16 (95% confidence interval (CI) 0.62–2.17) for the heterozygous mutant G/A, and 0.41 (95% CI 0.21–0.82) for the homozygous mutant G/G. There was also a significant difference for the polymorphism rs3219476 T/G (*cP* = 0.0036); the OR was 2.12 (95% CI 1.26–3.59) for the heterozygous mutant T/G and 3.81 (95% CI 1.81–8.04) for the homozygous mutant T/T. However, there was no significant difference between the RA patients and control individuals for rs3219489 or rs3219493. The differences in allele frequencies of these polymorphisms between individuals with and without RA were similar to the results of the genotypes frequencies (Table 2). There were significant differences in allele frequencies for the rs3219463 (*cP* = 0.0064, OR 0.63, and 95% CI 0.47–0.84) and rs3219476 polymorphism (*cP* = 0.0096, OR 1.56, and 95% CI 1.17–2.07), while the allele frequencies of the rs3219489 and rs3219493 polymorphisms were not significantly different (*cP* = 0.318 and 2.684, resp.). The dominant and recessive distributions of these two RA-related SNPs also presented significant differences of the frequencies, except for dominant distribution of rs319463. These results demonstrate a significant difference in the allele, genotype, dominant, and recessive distribution for the rs3219463 and rs3219476 polymorphisms between the RA and control groups.

### 3.3. Distribution of* MUTYH* Haplotypes

**Figure 1:**
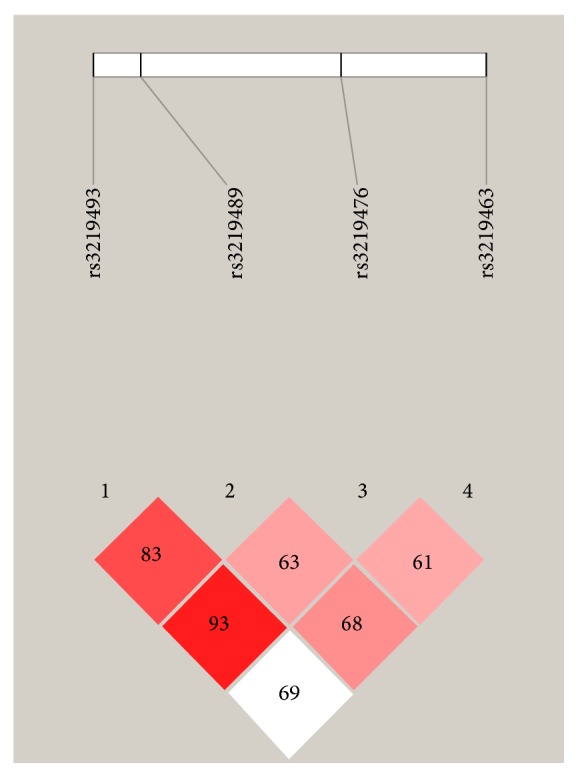
Four SNPs on the LD map pf* MUTYH* gene. LD coefficients (*D*′) between four SNPs of* MUTYH* gene were graphically indicated.

To further investigate whether haplotypes of* MUTYH *were correlated with RA, the LD map was constructed (Figure 1) and haplotype frequencies differences were estimated for the 4 identified polymorphisms. The pattern of LD in the MUTYH locus was measured by *D*′ score among the 4 SNPs. Linkage disequilibrium indicated that rs3219493 and rs3219489 had a high LD score (*D*′ > 80), and so did rs3219493 and rs3219476. However, rs3219463 and rs3219476 had a low LD score (*D*′ = 61).

Of the 12 observed haplotypes, haplotypes with frequency greater than 5% were presented (Table 3) and were further analyzed. After performing a haplotype-specific analysis, the haplotypes Ht6-GTGC and Ht8-GGCG demonstrated significantly lower frequencies in RA patients than in the control groups (Ht6-GTGC, *P* = 0.0076, OR 0.41, and 95% CI 0.21–0.81; Ht8-GGCG, *P* = 0.0070, OR 0.32, and 95% CI 0.13–0.76), while after Bonferroni correction, the differences were not significant. There were no differences in haplotype frequency between the RA and control groups for the other haplotypes.

### 3.4. *MUTYH* Polymorphisms and Clinical Features of RA

The association between the clinical features of patients with RA with the genotypes rs3219463 and rs3219476 was analyzed (Table 4). Among the patients with RA, the genotype frequency of rs3219463 G/− was significantly greater in patients with immunoglobulin M rheumatoid factors (RF) (*P* = 0.019, OR 2.70, and 95% CI 1.155–6.312). However, a comparison of the genotype frequency of rs3219476 and the clinical features revealed no significant difference among RA patients. There was no significant association between the haplotypes Ht6-GTGC and Ht8-GGCG and the clinical features among RA patients (Table 5).

## 4. Discussions

In the present study, we had found two SNPs of the* MUTYH* gene associated with RA. The genetic/allelic frequencies of rs3219463 (in the promoter region) and rs3219476 (in an intron) showed significant differences between RA and control patients. Our results provide the evidence for genetic association coordinated by these polymorphisms with the clinical features of RA. There have been some reports that showed the relationship between the DNA repair pathway and autoimmune diseases. For example, dysfunctional T cells show chronic inflammatory immune responses in the synovium of RA patients, and the DNA repair kinase* ATM* may be involved [6]. Furthermore, it has been postulated that SNPs in the* X-ray repair cross-complementing gene 1* (*XRCC1*), one of the BER proteins, may correlate with RA [21]. To our knowledge, this study is the first investigation to demonstrate that gene polymorphisms in* MUTYH*, which encodes a glycosylase member of BER, may contribute to the pathogenesis of RA.

ROS-associated base damage can be repaired by the BER pathway, which is initiated by many damage-specific glycosylases that cleave the glycosylic bond between the damaged base and the sugar phosphate backbone [8].* MUTYH*, which removes adenine paired with 8-oxo-G, is important in suppressing G·C to T·A transversions and in preventing CRC in people and mice. In murine studies,* MUTYH*-deficient mice were more susceptible to spontaneous tumors [22] and oxidative stress-induced intestinal tumors. MUTYH-associated polyposis (MAP), an autosomal recessive disorder, is associated with biallelic germline mutations in* MUTYH* [11, 23] and was first reported in 2002. Since then, many aspects of MAP have been investigated. The possibility that some mutations are nonpathogenic polymorphisms that are found coincidentally in patients with colorectal adenomas and carcinomas cannot be ruled out [8].

RA, a chronic inflammatory disease with tissue-destructive potential, is now recognized as a complex genetic disorder. Although numerous studies have reported an association between RA and HLA-DRB1*∗*04, cytokines, and CD4 cells, other genes may contribute to disease susceptibility [4]. Disease risk genes may increase the risk of pathological reactions, which suggests a gene-dose effect. One effect involves a snowballing mechanism that leads to increased levels of cellular damage and death resulting in more inflammation that induces the production of more ROS [9]. Several SNPs of various genes were found related to the risk of RA among Taiwanese or Chinese population. For example, an SNP (rs7135617) located in the intron region of* ORAI1* gene was associated with a risk of RA in the Taiwanese population [24]. The polymorphism of FCGR2B was related to early-onset of RA and the polymorphism of FCGR3A may influence RF production [25, 26]. Furthermore, there were many studies discussing the IL-family associated with pathogenesis of RA in Chinese population, such as IL-4, IL-6, and IL-18 [27, 28]. The question remains whether defects in DNA damage repair render individuals susceptible to RA or whether this is a consequence of the disease. The accumulation of DNA damage may also have broader implications on impairing diverse cellular functions.

In haplotype studies, 2 haplotypes of the* MUTYH* gene showed differences between the RA and control groups (Table 3). The haplotypes Ht6-GTGC and Ht8-GGCG had lower presenting rates in RA patients than in the control groups; these appeared to be “protective” haplotypes. The rs3219463 polymorphism positioned in the* MUTYH* gene promoter region; the rs3219476 and rs3219493 polymorphisms positioned in the* MUTYH* gene intron region; and rs3219489 polymorphism positioned in the* MUTYH* gene exon region (Gln338His). We found that investigation of the haplotype of the* MUTYH* gene gave an additional sign for determining whether this gene is associated with RA.

Previous studies have shown that patients with erosive RF-positive joint disease are characterized by the genotype HLA-DRB1*∗*4/RA nonassociated allele [4]. In this study, we found that the G/− frequency of rs3219463 was significantly increased in patients with RF (Table 3). Therefore, we postulated that rs3219463 may be a risk factor for RF in RA patients. How the RF factor contributes to the disease process is unknown, but it has been known to facilitate antigen presentation. Immune complexes of RF and immunoglobulin G may contribute to disease activity by activating complements and stimulating cytokine synthesis [1]. From a clinical perspective, the dissection of the phenotypic and genotypic variants of RA is critical for further exploration of targeted therapeutic treatment.

There were two major limitations in this study. Firstly, because of the noncompatibility in Hardy-Weinberg equilibrium of rs3219476 genotype distributions, we minimized errors in genotyping and validated our findings by repeating the genotyping analysis several times and obtaining consistent results. Therefore, occurrence of genotyping errors in this study was kept to a minimum. Moreover, the T/G genotype compared with TT genotype of rs3219476 has been revealed as a protective genotype of cholangiocarcinoma among individuals of Han Chinese living in Jiangsu province in China [29]. Based on the genotype frequencies in the controls, the *P* value is 0.0859, which is in Hardy-Weinberg equilibrium but very close to 0.05. Compared to our data, we regard the occurrence frequency of rs3219476 as consistent. Furthermore, we tried to trace the ancestry background of controls and patients and found that study subjects were a mixed population of Minnan, Hakka, and Canton descendants. According to the paper published by Pan et al. [30], they mentioned that the SNP profiles in the major histocompatibility complex (MHC) region (6p21.3) showed no significant difference among Minnan descendants, Hakka descendants, and mixed population of Minnan and Hakka descendants, which indicate the homogeneity of the population. Thus population stratification should not produce in this study [30].

Secondly, due to the limited subject number from one medical center in this study, many comparisons revealed no significant differences after Bonferroni correction. Besides, neither the genotype of rs3219463 and rs3219476 nor the haplotypes were significantly associated in patients with extra-articular RA erosion. However, we had evaluated the power in this sample size. For rs3219463 and rs3219476, the statistical powers were 72.1% and 69.9%, respectively. And these two SNPs represent low linkage disequilibrium (Figure 1). Thus, the* MUTYH* polymorphisms did associate with susceptibility of RA. Even so, our data may be biased by the relatively small number of subjects and larger-scale studies are needed to confirm our findings.

In conclusion, our findings demonstrate that the rs3219463 and rs3219476 polymorphisms in the* MUTYH* gene in patients with RA in a Taiwanese population were associated with disease susceptibility. Although the exact function of the* MUTYH* polymorphisms cannot be confirmed in this study, we suggest that genetic variations in the* MUTYH* gene may affect BER efficiency and play a role in the progression of RA.

## Supplementary Material

Supplementary Table: A case and control association study was performed. There are no significant differences in gender and age between control subjects and RA patients.

## Figures and Tables

**Table 1:** Primers and PCR conditions used in this study to determine *MUTYH* gene polymorphisms.

Gene	SNP rs number	Position	Alleles	Primers	PCR product size (bp)	PCR conditions (annealing temperature)	Restriction enzyme site	DNA fragment size (bp)
*MUTYH *	rs3219463	1:45579019 (promoter region)	A/G	Forward: 5′-TCCCACAAGCCTTTGTAACC-3′ Reverse: 5′-CGTATTGGGAGCTCTGGTGT-3′	347	60°C	SbfI	A: 347 G: 161 + 186
	rs3219476	1:45575257 (intron)	G/T	Forward: 5′-GTTGCAGTGAGCCAAGATCA-3′ Reverse: 5′-AACCTGCCTTTTGTGAGCTG-3′	311	58°C	BtsCI	G: 58 + 172 + 81 T: 58 + 253
	rs3219489	1:45570092 (exon)	G/C	Forward: 5′-TGTGGTGAGCACCAAACCTA-3′ Reverse: 5′-GGCTGTTCCAGAACACAGGT-3′	319	62°C	HpyCH4III	G: 193 + 126 C: 319
	rs3219493	1:45568856 (intron)	G/C	Forward: 5′-CTCAAGTGATCCACCCGACT-3′ Reverse: 5′-AGTGAAGCCTGGAGTGGAGA-3′	332	58°C	HpyCH4V	G: 332 C: 139 + 193

**Table 2:** Association between genotypic and allelic distributions of *MUTYH* gene polymorphisms and individuals with RA.

SNP database ID	RA number (%)	Controls number (%)	*P* value/*cP* value	Odds ratio (95% CI)
rs3219463				
G/G	37 (19.3)	76 (39.6)	0.00006^*^/	0.41 (0.21–0.82)
G/A	129 (67.2)	94 (49.0)	0.00024^*^	1.16 (0.62–2.17)
A/A	26 (13.5)	22 (11.4)		1.00
G allele	203 (52.9)	246 (64.1)	0.0016^*^/	0.63 (0.47–0.84)
A allele	181 (47.1)	138 (35.9)	0.0064^*^	1.00
G/G + G/A	166 (86.5)	170 (88.5)	0.5371/	0.83 (0.45–1.52)
A/A	26 (13.5)	22 (11.5)	2.1496	1.00
G/G	37 (19.3)	76 (39.6)	0.00001^*^/	0.36 (0.23–0.58)
G/A + A/A	155 (80.7)	116 (60.4)	0.00006^*^	
rs3219476				
T/T	33 (17.2)	17 (8.9)	0.0009^*^/	3.81 (1.81–8.04)
T/G	132 (68.8)	122 (63.5)	0.0036^*^	2.12 (1.26–3.59)
G/G	27 (14.0)	53 (27.6)		1.00
T allele	198 (51.6)	156 (40.6)	0.0024^*^/	1.56 (1.17–2.07)
G allele	186 (48.4)	228 (59.4)	0.0096^*^	1.00
T/T + T/G	165 (86)	139 (72.4)	0.0011^*^/	2.33 (1.39–3.90)
G/G	27 (14.0)	53 (27.6)	0.0044^*^	1.00
T/T	33 (17.2)	17 (8.9)	0.013^*^/	2.17 (1.17–4.05)
T/G + G/G	159 (82.8)	178 (91.1)	0.0650	1.00
rs3219489				
G/G	52 (27.1)	70 (36.5)	0.1383/	0.61 (0.33–1.13)
G/C	106 (55.2)	94 (49.0)	0.5532	0.93 (0.52–1.65)
C/C	34 (17.7)	28 (14.5)		1.00
G allele	210 (54.7)	234 (60.9)	0.0795/	0.77 (0.58–1.03)
C allele	174 (45.3)	150 (39.1)	0.3180	1.00
G/G + G/C	158 (82.3)	164 (85.5)	0.4053/	0.79 (0.46–1.37)
C/C	34 (17.7)	28 (14.5)	1.6212	1.00
G/G	52 (27.1)	70 (36.5)	0.0485^*^/	0.65 (0.42–1.00)
G/C + C/C	140 (72.9)	122 (63.5)	0.1940	1.00
rs3219493				
G/G	164 (85.4)	161 (83.9)	0.671/	1.13 (0.65–1.97)
G/C	28 (14.6)	31 (16.1)	2.6840	1.00
C/C	0 (0)	0 (0)		
G allele	356 (92.7)	353 (91.9)	0.6844/	1.12 (0.66–1.90)
C allele	28 (7.3)	31 (8.1)	2.7376	1.00
G/G	164 (85.4)	161 (83.9)	0.6712/	1.13 (0.65–1.97)
G/C + C/C	28 (14.6)	31 (16.1)	1.3424	1.00

95% CI: 95% confidence intervals.

^*∗*^ *P* value of <0.05 was determined as statistically significant. The chi-square test was performed to obtain the *P* value.

^*∗*^ *cP* value: *P* value corrected by Bonferroni correction.

**Table 3:** Odds ratios and 95% confidence intervals for the association between *MUTYH* gene haplotypes and RA.

Haplotype	rs3219463	rs3219476	rs3219489	rs3219493	RA, %	Control, %	*P* value/*cP* value	Odds ratio (95% CI)
Ht1	G	G	G	G	150 (39.1)	173 (45.1)	0.0927/0.7416	0.78 (0.59–1.04)
Ht2	A	T	C	G	124 (32.3)	102 (26.6)	0.0815/0.652	1.32 (0.97–1.80)
Ht3	G	T	C	G	19 (4.9)	11 (2.9)	0.1362/1.0896	1.77 (0.83–3.76)
Ht4	A	G	C	G	17 (4.4)	15 (3.9)	0.718/5.744	1.14 (0.56–2.32)
Ht5	G	T	G	G	13 (3.4)	12 (3.1)	0.8389/6.7112	1.09 (0.49–2.41)
Ht6	G	T	G	C	13 (3.4)	30 (7.8)	0.0076^*^/0.0608	0.41 (0.21–0.81)
Ht7	A	G	G	G	11 (2.9)	19 (4.9)	0.1362/1.0896	0.57 (0.27–1.21)
Ht8	G	G	C	G	7 (1.8)	21 (5.5)	0.007^*^/0.056	0.32 (0.13–0.76)

95% CI: 95% confidence intervals.

^*∗*^ *P* value of <0.05 was determined as statistically significant.

The chi-square test was performed to obtain the *P* value.

^*∗*^ *cP* value: *P* value corrected by Bonferroni correction.

**Table 4:** Frequencies of rs3219463 genotypes of RA patients with various clinical features.

rs3219463 genotype	RF	Extra-articular	Erosion	rs3219476 genotype	RF	Extra-articular	Erosion
	*n* (%)	*n* (%)	*n* (%)		*n* (%)	*n* (%)	*n* (%)
G/− (*n* = 166)	126 (75.9)	81 (48.8)	83 (50.0)	T/− (*n* = 165)	119 (72.1)	75 (45.5)	87 (52.7)
A/A (*n* = 26)	14 (53.8)	10 (38.5)	14 (53.8)	G/G (*n* = 27)	22 (81.5)	16 (59.3)	10 (37.0)
*P *	0.019^*^	0.326	0.715	*P*	0.307	0.183	0.131
OR	2.700	1.525	0.857	OR	0.588	0.573	1.896
95% CI	1.155–6.312	0.654–3.556	0.374–1.964	95% CI	0.21–1.65	0.25–1.31	0.82–4.39
A/− (*n* = 155)	109 (70.3)	76 (49.0)	79 (51.0)	G/− (*n* = 159)	120 (75.5)	77 (48.4)	77 (48.4)
G/G (*n* = 37)	31 (83.8)	15 (40.5)	18 (48.6)	T/T (*n* = 33)	21 (63.6)	14 (42.4)	20 (60.6)
*P *	0.098	0.353	0.800	*P*	0.161	0.530	0.203
OR	0.459	1.411	1.097	OR	1.758	1.274	0.610
95% CI	0.179–1.174	0.681–2.2922	0.535–2.249	95% CI	0.79–3.90	0.60–2.72	0.28–1.31

95% CI: 95% confidence intervals. OR: odds ratio.

^*∗*^ *P* value of <0.05 was determined as statistically significant. The chi-square test was performed to obtain the *P* value.

RF: rheumatoid factors.

**Table 5:** Frequencies of *MUTYH* haplotype (Ht7) of RA patients with various clinical features.

Ht6 genotype	RF	Extra-articular	Erosion	Ht8 genotype	RF	Erosion
	*n* (%)	*n* (%)	*n* (%)		*n* (%)	*n* (%)
Ht6/non-ht6 (*n* = 13)	10 (76.9)	7 (53.8)	9 (69.2)	Ht8/non-ht8 (*n* = 7)	6 (85.7)	3 (42.9)
Non-ht6/non-ht6 (*n* = 179)	130 (72.6)	84 (46.9)	88 (49.2)	Non-ht8/non-ht8 (*n* = 185)	134 (72.4)	94 (50.8)
*P *	0.736	0.630	0.162	*P*	0.438	0.679
OR	1.256	1.319	2.327	OR	2.284	0.726
95% CI	0.33–4.76	0.43–4.08	0.69–7.83	95% CI	0.27–19.44	0.16–3.33

95% CI: 95% confidence intervals. OR: odds ratio.

RF: rheumatoid factors.
