# Supplementary material for: MUTYH Gene Polymorphisms as Risk Factors for Rheumatoid Arthritis
Source: Biomed Res Int. 2015 Jul 27;2015:893796. doi: 10.1155/2015/893796 (PMC4530216; doi:10.1155/2015/893796)
Supplement: Supplementary file 1 [file 893796.f1.docx]

Supplementary Table 1: The demographic characteristics of all subjects.

Variables	Case (n = 192)	Control (n = 192)
Sex (female/male)	151 / 41	151 / 41
Age (years)	53.4 ± 13.7	50.5 ± 12.9
